# Correlation between Cigarette Smoking and Urine Cotinine Level in Gastric Cancer Patients

**Published:** 2014

**Authors:** Nima Babhadiashar, Masoud Sotoudeh, Ebrahim Azizi, jafar Bashiri, Reza Didevar, Reza Malekzadeh, Mohammad Hossein Ghahremani

**Affiliations:** a *Department of Pharmacology and Toxicology, Faculty of Pharmacy, Tehran University of Medical Sciences, Tehran, Iran.*; b *Department of Pathology, Shariati Hospital, Tehran University of Medical Sciences, Tehran, Iran. *; c *Digestive Diseases Research Institute, Shariati Hospital, Tehran University of Medical Sciences, Tehran, Iran.*; d *Department of Pathology Ardabil Branch, Islamic Azad University, Ardabil, Iran.*

**Keywords:** Gastric cancer, Cigarette, Smoking, Cotinine, Urine

## Abstract

Various substances in cigarette smoke including nicotine have been shown to promote/induce cancer cell proliferation. Since cotinine has a longer half life and stability in the blood, it has become the preferred biomarker for cigarette smoking exposure.

Seventy-three gastric cancer patients were included in this study. The tumor tissues were stained with H & E for pathological evaluation. The cotinine levels were measured in urine using a competitive ELISA. Tumors were 90% adenocarcinoma with 63% intestinal and 37% diffuse subtypes. Tumors were poorly (45.2%) or moderately differentiated (41.1%) and localized mainly (77%) in the upper part of stomach. The levels of cotinine were significantly different between smoker (283.83 ± 178.10 ng/mL) and non-smoker (39.28 ± 113.34 ng/mL) groups (p < 0.001). However, there is no-significant correlation between tumor characteristics and cotinine level in smoker patients.

Cotinine level correlates with smoking in gastric patients, however, correlation with the tumor features has not been observed.

## Introduction

Gastric carcinoma is one of the common and fatal diseases worldwide (1, 2). In Europe, gastric cancer is ranked sixth following lung, breast, colorectal, prostate and bladder cancers (2, 3). Nearly 60% of gastric cancer cases are from developing countries; however, the disease incidence differs among races (1). The frequency of gastric cancer remains high in Iran and renders gastric carcinoma as one of common fatal cancers with nearly 7300 new cases every year (1). The highest prevalence is the Ardabil province (1, 4). 

About 95% of all malignant gastric tumors are adenocarcinoma (2). One of the basic classifications based on tumor histology is by Lauren (5, 6), which divides gastric cancers into intestinal or diffuse types. Many risk factors including smoking, antioxidant deficiency and high salt intake are involved in the pathogenesis of stomach cancer (7, 8). Assessments of the relation between gastric cancer and smoking have revealed that about 18% of gastric cancer cases are attributed to tobacco smoking (9-11). A Patient smoking history as an exclusive parameter for evaluation of smoking status is unreliable, therefore, as a reliable consideration; biochemical analysis is required (12). In this regard, the level of nicotine and its metabolites in body fluids can translate the smoking status of patient (12-14). Nicotine, the major compound in cigarette smoke, is absorbed by the lungs and metabolized to several different derivatives (14-16). Although there are various methods to measure nicotine in smokers (17, 18), its elimination half life of is short (19). Cotinine is the major metabolite of nicotine with a longer half life (about 20 h) than nicotine (about 20-60 min) (14, 16 and 17). Besides, cotinine has higher urinary and serum concentrations, makes cotinine as suitable biomarker for screening tests of cigarette smokers (15, 16). Various methods have been developed for measuring urine cotinine including Enzyme-Linked Immuno absorbent Assay (ELISA), Radioimmunoassay, Liquid chromatography, Gas chromatography and HPLC (20, 21). Among them, ELISA is easy to set up, fast and does not need time-consuming biological sample preparation (23, 24). In this study we have measured cotinine in gastric cancer patients and correlated it to smoking history and tumor location.

## Experimental

This study was conducted in outpatient gastrointestinal disease clinic in Ardabil city and Masoud clinic in Tehran after obtaining an informed consent from the patients. During 18 months period (Jan 2009-Aug 2010), seventy-three patients who had gastrointestinal symptoms/signs such as abdominal pain, weight loss, dyspepsia, anorexia, nausea and vomiting were subjected to standard upper GI endoscopy. At least six biopsies were obtained from all detected tumors. All biopsies were fixed in 10% buffered formalin, sectioned and stained by the hematoxylen-eosin technique then observed by experienced pathologists under microscope for cancerous tissues. A structured questionnaire consisting of demographic characteristics, past disease history, drug history, family disease history, smoking habits (cigarettes/day), radiotherapy and chemotherapy was completed for patients. Five milliliters of mid-stream urine were collected in special vessels and were stored at -20 °C until cotinine measurement.

*Histochemistry*

The tumor samples were parafinized for further pathological evaluation. Several sections with three micrometers thickness from each block were used to perform hematoxylen-eosin staining. 

According to the standard protocol, all sections were dewaxed in the xylene, rehydrated using a sequence of decreasing concentration of alcoholic solutions. The slides then immersed in hematoxylene and acid alcohol solutions, respectively. Slides were rinsed with tap water and submerged in eosin for one minute. After washing, the slides were dehydrated by increasing concentration of alcoholic solutions and were cleaned by xylene. Cover-slip mounted on the labeled glass slide with mounting media for microscopic observation. Two independent pathologists observed all slides and data were collected.

*Cotinine measurement*

The cotinine ELISA kit (Calbiotech, U.S.A) is a competitive assay based on Horse Radish Peroxidase conjugated antibody to detection the cotinine in the body liquids. At the first, 10 µL of standards or urine specimens and 100 µL of enzyme conjugate were added to each well in duplicate and incubated for 60 minutes in room temperature (RT) at dark. Then wells washed six times with 300 µL of distilled water, and 100 µL of substrate was added and incubated for 30 minutes at the dark and RT condition. Finally the reaction was stopped by adding 100 µL of stop solution to each well and the absorbance was measured at the wavelength of 450 nm. 

*Statistical analysis*

Descriptive data are presented as mean and standard deviation (SD) or percentages. We used linear regression model and Box-Cox transformation to calculate cotinine concentrations from OD (Optical Density) values obtained from ELISA kit using the following formulate.

$\mathrm{cotinine concentration}={\left(\frac{\frac{1}{\sqrt[{2}]{\mathrm{OD}}}-0.428}{0.179}\right)}^{2}$

Mann-Whitney and Chi-Square tests were used to evaluate correlation between cotinine concentrations and demographic characteristics, in smokers and non-smokers groups. 

## Results

*Characteristics of Patients*

Table 1 summarizes the distribution of seventy-three gastric cancer patients by demographic characteristics and smoking status. Of the seventy-three cases of gastric cancer patients, 51 were male and 22 were female (Mean age: 65.12 ± 13.177 Yr, Range: 25-88 Yr). Non smoker group consists of 33 male and 21 female and smoker group includes 19 male and 1 female patients. The mean age, weight and height in both groups had no-significant difference and were matched (Table1).

**Table 1:** The descriptive information of gastric patients

	**Total**	**Mean**	**SD**	**Median**	**Min.**	**Max.**
**Age**	73	65.12		67	25	88
Non-Smoker	53	64.84	2.06	68.5	25	87
Smoker	20	63.76	3.24	62	39	88
**Height**	73	166.15		168	150	182
Non-Smoker	53	165.2	1.01	166.5	150	178
Smoker	20	168.71	1.77	170	155	182
**Weight**	73	67.4		66	40	94
Non-Smoker	53	68	1.77	66.5	40	94
Smoker	20	65.94	2.75	65	40	90
**Smoking Status **						
Duration of Smoking	20	34.76	12.45	38	12	56
Average # of Cig./Day	20	18.93	8.22	20	4	40
Average # of Pack/Year	20	345.47	150.08	365	73	730

Average cigarette per day in smokers was 18.93 ± 8.224 (Range: 4-40) and they were smoking for 34.76 ± 12.45 years (Range: 12-56). The average calculated pack/years in patients of this study was 345.47 ± 150.08 (Range: 73-730 Pack/Year).

*Tumor description*

Tumors were 90% adenocarcinoma with intestinal type in 48 (63%), diffused type in 25 (37%) samples. Based on the pathological evaluations, there were 1 undifferentiated (1.4%), 33 poorly differentiated (45.2%), 30 moderately differentiated (41.1%) and 5 well-differentiated (6.8%) tumors.

**Figure 1:**
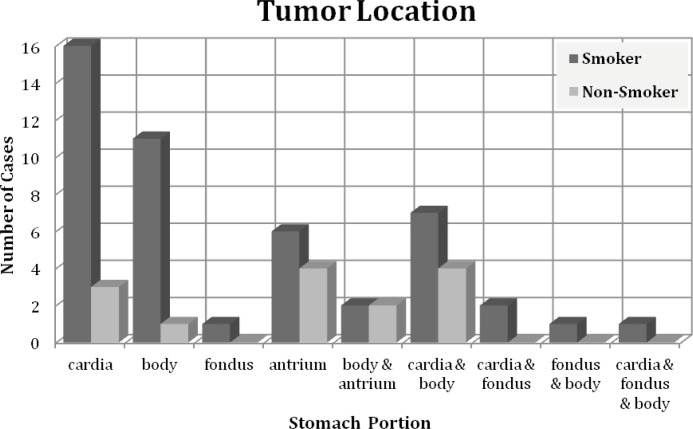
The location of gasteric tumor in smoking and non-smoking patients

Gastric tumors are usually divided into cardia and non-cardia tumors, based on pathogenesis. We have used gastric sub divisions (Cardia, Body, Fundus, Antrium and Pylor) for tumor location. Our results indicated that in smoker group tumors were mainly located in Cardia, body and antrium. On the other hand, in non smoker group tumors were located mostly in antrium and cardia (Figure 1). Interestingly, tumors were localized in both cardia and body in 7 patients in smoking and 4 patients in non-smoking groups (Figure 1).Tumors in more than one location were found in 1/3 of patient in smoking group compared to non-smoking patients (Figure 1). Moreover, fundus was not involved in non-smoking patients. 

*Cotinine level in patients*

**Figure 2:**
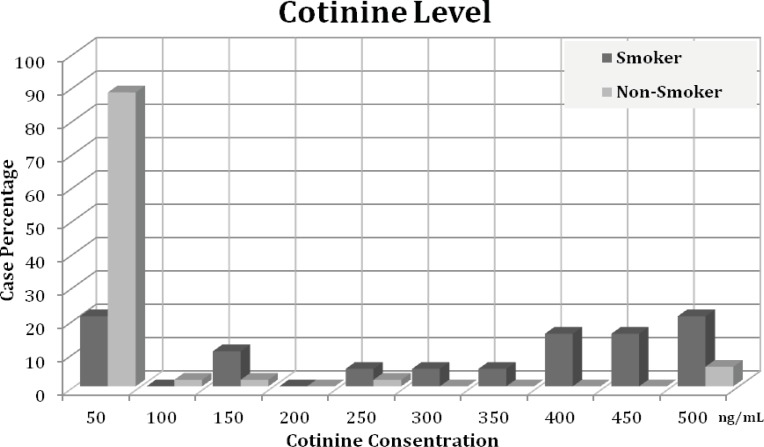
The distribution of cotinine level in smoking and non-smoking patients

The results indicate that the cotinine level ranging is from 0.14 to 478.54 ng/mL (Figure 2). The cotinine level in most of the non-smoker group was lower than 50 ng/mL; there were some cases with high levels of cotinine. Mean Cotinine urine concentrations in smokers and non-smokers were 39.28 ± 113.33 and 283.83 ± 178.10, respectively. The statistical analysis indicated a significant difference between cotinine levels in two groups (p < 0.001). However, there were no significant correlation between cotinine levels and average number of cigarettes (Spearman r = 0.061) or duration of smoking (Spearman r = -0.038). The effect of age on cotinine level in smoking group was non-significant (P = 0.1) and no-correlation between cotinine level and tumor type in smoker patients was found (p = 0.89).

## Discussion

Our assessment of level of urine cotinine showed that there is a significance difference between smokers and none smokers in cotinine level. We could not find significant correlation between smoking status (number and duration of smoking) and urine cotinine concentration. The Cotinine production can be affected by metabolic enzyme polymorphism in patients. It has been reported that polymorphism of CYP2A6 can affect the level of cotinine in patients as well as the number of smoked cigarettes (24-26). Moreover, polymorphism in UGT2B10 changes the glucorinidation of cotinine and nicotine, thus change the level of these compound in urine of patients (27).

Thus, considering enzyme polymorphism will better correlate smoking status and cotinine level. However, the cotinine level is significantly different from non-smoking group. Besides, we have observed high levels of cotinine observed in some of the non-smokers. Further analysis showed that this high level is due to their exposure to second hand smoke.

Evaluation of tumor pathology and cotinine level indicated that there is not a correlation between cotinine level and the tumor status. However, a trend toward multiple tumors was observed in smoking group. A recent cohort study showed that smokers with serum cotinine level less than 55 nmol/mL have low risk of pancreatic cancer (28). 

The effects of nicotine on gastrointestinal conditions are probably due to a number of local and systemic actions. These actions, in combination with other important etiologic factors, could be responsible for disease outcome. Thus, level of cotinine may be correlated with the gastric tumors. However, more detailed analyses are required to confirm the correlation. 
